# Predicting the Dispersion Relations of One-Dimensional Phononic Crystals by Neural Networks

**DOI:** 10.1038/s41598-019-51662-3

**Published:** 2019-10-25

**Authors:** Chen-Xu Liu, Gui-Lan Yu

**Affiliations:** School of Civil Engineering, Beijing Jiaotong University, Beijing, 100044 China

**Keywords:** Computational science, Composites

## Abstract

In this paper, deep back propagation neural networks (DBP-NNs) and radial basis function neural networks (RBF-NNs) are employed to predict the dispersion relations (DRs) of one-dimensional (1D) phononic crystals (PCs). The data sets generated by transfer matrix method (TMM) are used to train the NNs and detect their prediction accuracy. In our work, filling fractions, mass density ratios and shear modulus ratios of PCs are considered as the input values of NNs. The results show that both the DBP-NNs and the RBF-NNs exhibit good performances in predicting the DRs of PCs. For one-parameter prediction, the RBF-NNs have shorter training time and remarkable prediction accuracy, for two- and three-parameter prediction, the DBP-NNs have more stable performance. The present work confirms the feasibility of predicting the DRs of PCs by NNs, and provides a useful reference for the application of NNs in the design of PCs and metamaterials.

## Introduction

A composite structure in which elastic constants are periodically distributed is called a phononic crystal (PC). When an elastic wave is periodically modulated by the elastic constant, a phonon bandgap may be generated, and the propagation of elastic waves in a certain frequency range is suppressed or prohibited. This bandgap characteristics opened a new prospect in wave control and manipulations^1^.

In dealing with the calculation of PCs, especially with the structure optimization, the traditional calculation methods, such as transfer matrix method (TMM), plane wave expansion method, finite element method, etc., consume a lot of time and computer memory.

Benefiting from the concept of deep learning method^2^, artificial intelligence technology has ushered in a rapid development in the past few years. As an important member of artificial intelligence technology, neural network (NN) plays an important role in all walks of life, owing to its massively parallel distributed structure of NN and its superior learning ability. Google’s “Alpha Go”, autopilot technology, face recognition technology, text translation technology and medical automatic diagnosis technology, etc., are typical examples of NN applications.

PCs were proposed by analogy to photonic crystals^3–6^, but are more complex because of the vector property of elastic waves. Few literatures have been mentioned so far involving the application of NNs in PCs except for the work by Finol *et al*.^7^ who compared the prediction accuracy of multilayer perceptron and convolutional neural network (CNN) for one-dimensional (1D) PCs; meanwhile, he used CNN to predict the eigenvalues of two-dimensional (2D) PCs. However, in the field of photonic crystal, there have been some preliminary results. Adriano *et al*.^8,9^ used a multilayer perceptron and an extreme learning machine to identify the bandgap width of 2D photonic crystals and the dispersion curves of 2D and three-dimensional (3D) photonic crystals. Christian *et al*.^10^ used a NN with a hidden layer to identify the crystalized size of the zinc oxide quantum dots and the energy of the band gap. Liu *et al*.^11^ achieved the inverse design of optical wave metamaterials by generative adversarial networks. Dong *et al*.^12^ compared the performance of three convolutional NNs and a support vector machine to identify the photonic bandgap width of a 2D “graphene-boron nitride” periodic structure.

This study aims to predict the DRs of 1D PCs and reduce the calculation cost by means of NNs. Two NN models, deep back propagation neural network (DBP-NN) and radial basis function neural network (RBF-NN)^13,14^, are trained, and their performances are tested. The investigation has been carried out for three cases, that are respectively one-, two- and three-parameter prediction. The present work is hopeful to provide an effective method for the analysis of PCs and to lay a foundation for the intelligent inverse design of PCs and metamaterials.

## Problem Description

**Figure 1:**
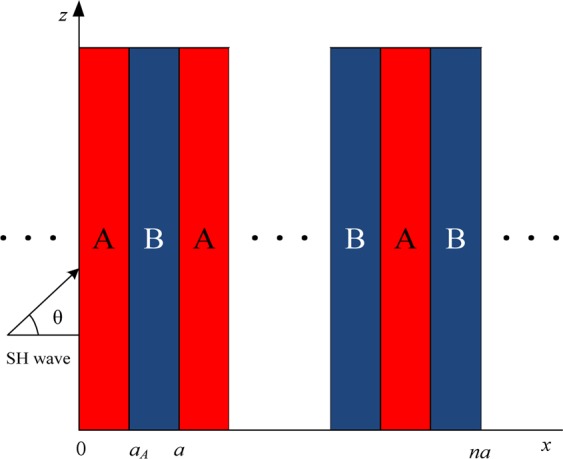
One-dimensional phononic crystal.

Consider shear-horizontal (SH) waves propagate in a 1D PC shown in Fig. 1. Material A and B are periodically arranged in *x*-direction with $\,a={a}_{A}+{a}_{B}$, where *a* is a lattice constant; *a*_*A*_ and *a*_*B*_ are, respectively, the thickness of material A and B in one-unit cell; and *θ* denotes the incident direction of SH waves.

It is known that the TMM has advantages in dealing with 1D PCs. When the SH wave is obliquely incident on the 1D PCs, its governing equation can be expressed as

1 $$\rho \frac{{\partial }^{2}u(x,z,t)}{\partial {t}^{2}}=\mu (\frac{{\partial }^{2}u(x,z,t)}{\partial {x}^{2}}+\frac{{\partial }^{2}u(x,z,t)}{\partial {z}^{2}}),$$

where $u(x,z,t)$ is displacement of the PCs in the *y* direction, *μ* is shear modulus, $\rho$ is mass density, and *t* is time.

When a harmonic plane wave is considered, it can be assumed

2 $$u(x,z,t)=U(x){e}^{{\rm{i}}{k}_{z}z-{\rm{i}}\omega t},$$

where $\omega$ is angular frequency, and *k*_*z*_ is wave number of the SH wave in the *z* direction, which is a constant from the Snell theorem. Substituting Eq. (2) into Eq. (1), the displacement and the stresses of the material A and the material B in the *n*-th lattice are respectively

3a $${u}^{ni}(x,z,t)={e}^{{\rm{i}}{k}_{z}z-{\rm{i}}\omega t}({C}_{ni}^{tr}{e}^{{\rm{i}}{\alpha }_{i}x}+{C}_{ni}^{r}{e}^{-{\rm{i}}{\alpha }_{i}x}),\,i=A,B$$

3b $${\tau }_{xy}^{ni}(x,z,t)={\mu }_{i}{\alpha }_{i}{e}^{{\rm{i}}{k}_{z}z-{\rm{i}}\omega t}({C}_{ni}^{tr}{e}^{{\rm{i}}{\alpha }_{i}x}-{C}_{ni}^{r}{e}^{-{\rm{i}}{\alpha }_{i}x}),\,i=A,B$$

where, for $i=A$, $(n-1)a\le x\le (n-1)a+{a}_{A}$, for $i=B$, $(n-1)a+{a}_{A}\le x\le na$, and

3c $${\alpha }_{i}=\sqrt{{(\frac{\omega }{{c}_{i}})}^{2}-{k}_{z}^{2}},\,i=A,B$$

*c*_*i*_ and *α*_*i*_ ($i=A,B$) are the wave velocity and the wave vector components in *x* direction respectively, and ${C}_{ni}^{tr}$ and ${C}_{ni}^{r}$ ($i=A,B$) indicate the amplitudes of the transmitted and reflected waves of the material *i* in the *n*-th lattice respectively.

The displacement and stress at the interface between material A and material B are continuous, so when $x=(n-1)a$, ${u}^{nA}={u}^{(n-1)B}$ and ${\tau }_{xy}^{nA}={\tau }_{xy}^{(n-1)B}$, and when $x=(n-1)a+{a}_{A}$, ${u}^{nA}={u}^{nB}$ and ${\tau }_{xy}^{nA}={\tau }_{xy}^{nB}$.

Set

4a $${{\boldsymbol{\Psi }}}_{{\boldsymbol{ni}}}={[{C}_{ni}^{tr},{C}_{ni}^{r}]}^{{\rm{T}}},\,i=A,B$$

4b $${{\boldsymbol{H}}}_{{\bf{1}}}=[\begin{array}{cc}1 & 1\\ {\mu }_{A}{\alpha }_{A} & -\,{\mu }_{A}{\alpha }_{A}\end{array}],$$

4c $${{\boldsymbol{K}}}_{{\bf{1}}}=[\begin{array}{cc}{e}^{i{\alpha }_{B}a} & {e}^{-i{\alpha }_{B}a}\\ {\mu }_{B}{\alpha }_{B}{e}^{i{\alpha }_{B}a} & -\,{\mu }_{B}{\alpha }_{B}{e}^{-i{\alpha }_{B}a}\end{array}],$$

4d $${{\boldsymbol{H}}}_{{\bf{2}}}=[\begin{array}{cc}{e}^{i{\alpha }_{A}{a}_{A}} & {e}^{-i{\alpha }_{A}{a}_{A}}\\ {\mu }_{A}{\alpha }_{A}{e}^{i{\alpha }_{A}{a}_{A}} & -\,{\mu }_{A}{\alpha }_{A}{e}^{-i{\alpha }_{A}{a}_{A}}\end{array}],$$

4e $${{\boldsymbol{K}}}_{{\bf{2}}}=[\begin{array}{cc}{e}^{i{\alpha }_{B}{a}_{A}} & {e}^{-i{\alpha }_{B}{a}_{A}}\\ {\mu }_{B}{\alpha }_{B}{e}^{i{\alpha }_{B}{a}_{A}} & -\,{\mu }_{B}{\alpha }_{B}{e}^{-i{\alpha }_{B}{a}_{A}}\end{array}],$$

The relationship between the amplitudes of the incident and the reflected waves in the *n*-th lattice and the (*n* − 1)-th lattice can be obtained from Eq. (4), that is

5 $${{\boldsymbol{\Psi }}}_{{\boldsymbol{nB}}}={\boldsymbol{T}}{{\boldsymbol{\Psi }}}_{({\boldsymbol{n}}-{\bf{1}}){\boldsymbol{B}}},$$

where ***T*** is the transfer matrix, and ${\boldsymbol{T}}={{\boldsymbol{K}}}_{2}^{-1}{{\boldsymbol{H}}}_{2}{{\boldsymbol{H}}}_{1}^{-1}{{\boldsymbol{K}}}_{1}$.

Due to the periodicity in the *x* direction, use the Bloch theorem to get

6 $${{\boldsymbol{\Psi }}}_{{\boldsymbol{nB}}}={e}^{{\rm{i}}ka}{{\boldsymbol{\Psi }}}_{({\boldsymbol{n}}-{\bf{1}}){\boldsymbol{B}}},$$

where *k* is a 1D Bloch wave vector.

Substituting Eq. (5) into Eq. (6) gives a standard matrix eigenvalue problem, that is

7 $$|{\boldsymbol{T}}-{e}^{{\rm{i}}ka}{\boldsymbol{I}}|=0,$$

where *I* is a 2 × 2 unit matrix.

By solving the eigenvalues of the matrix ***T***, the DRs between the wave vector *k* and the angular frequency $\omega$ can be obtained^14^

8a $$\begin{array}{rcl}\cos (ka) & = & \cosh ({\alpha }_{A}{a}_{A})\cosh ({\alpha }_{B}{a}_{B})\\  &  & +\,\frac{1}{2}(F+\frac{1}{F})\sinh ({\alpha }_{A}{a}_{A})\sinh ({\alpha }_{B}{a}_{B}),\end{array}$$

8b $$F=\frac{{\alpha }_{A}{\rho }_{A}{c}_{A}^{2}}{{\alpha }_{B}{\rho }_{B}{c}_{B}^{2}},$$

**Figure 2:**
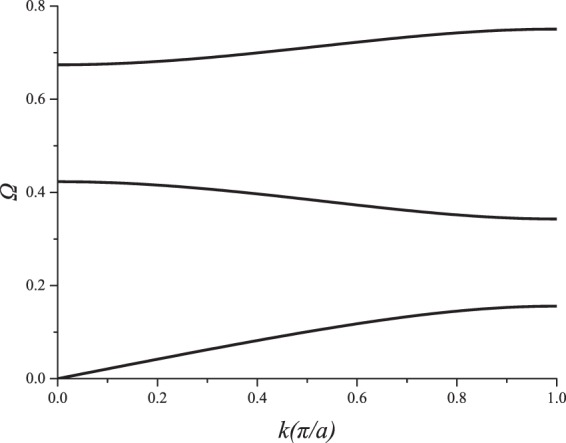
Dispersion relations.

If the material A and B are chosen, respectively, as aluminum and epoxy; ${a}_{A}={a}_{B}=0.075\,{\rm{m}}$, and $\theta =0.5\,{\rm{rad}}$; the DRs of a PC, i.e., the relations between eigen frequency and wave vector, can be obtained according to Eq. (8). Figure 2 gives the first three eigen modes of the PC, where $\Omega =\omega a/2\pi {c}_{A}$, is the normalized frequency.

From Eq. (8), we can see, the DRs of 1D PCs are determined by ${a}_{i}$, ${\mu }_{i}$ and ${\rho }_{i}(i=A,B)$ for a certain incident wave and periodic constant. Considering that for anti-plane waves, the shear modulus ratio, $\bar{\mu }={\mu }_{B}/{\mu }_{A}$, and the mass density ratio, $\bar{\rho }={\rho }_{B}/{\rho }_{A}$ are the main physical parameters affecting the PC bandgap^15^, and the filling fraction, $\bar{a}={a}_{B}/a$, is the key geometrical parameter. Hence, for our problem, we focus on the study of parameters of $\bar{\mu }$, $\bar{\rho }$ and $\bar{a}$ respectively. Thus, the relationship between DRs and PCs parameters can be written as

9 $$drs={f}_{a}(\bar{a},\bar{\mu },\bar{\rho }),$$

where *drs* represents the DRs, and *f*_*a*_ is the function which reflects the analytic relations between $\bar{a}$, $\bar{\mu }$, $\bar{\rho }$ and *drs*. The function *f*_*a*_ belongs to the category of transcendental equations, so it is difficult to get analytical solutions.

In this paper, our aim is training NNs to learn the relationship between DRs and PCs parameters or, in other words, making NNs simulate the function *f*_*a*_. The simulating relationship is written as

10 $$drs={f}_{s}(\bar{a},\bar{\mu },\bar{\rho }),$$

where *f*_*s*_ is the function which simulates the function *f*_*a*_. The ultimate goal of our work is obtaining the function *f*_*s*_ by NN.

We investigate this simulating relationship between DRs and PCs parameters for three cases. First, we investigate the relationship between $\bar{a}$ and DRs, then the relationship between $\bar{\mu }$, $\bar{\rho }$ and DRs, and finally, the relationship between $\bar{a}$, $\bar{\mu }$, $\bar{\rho }$ and DRs.

## Neural Networks and Data Set

In our work, the key issue is to get an input-output relationship by NN, where the input is the PCs parameters, namely, $\bar{a}$, $\bar{\mu }$ and $\bar{\rho }$, and the output is the DRs. NN is a technology driven by data. It can learn the features of input-output through being fed enough data. The NN returned from learning can predict the corresponding output, if an input is given, which is never learned by the NN. We are going to use enough data to train NNs for making them simulate the relationship between DRs and PCs parameters.

### DBP-NN

**Figure 3:**
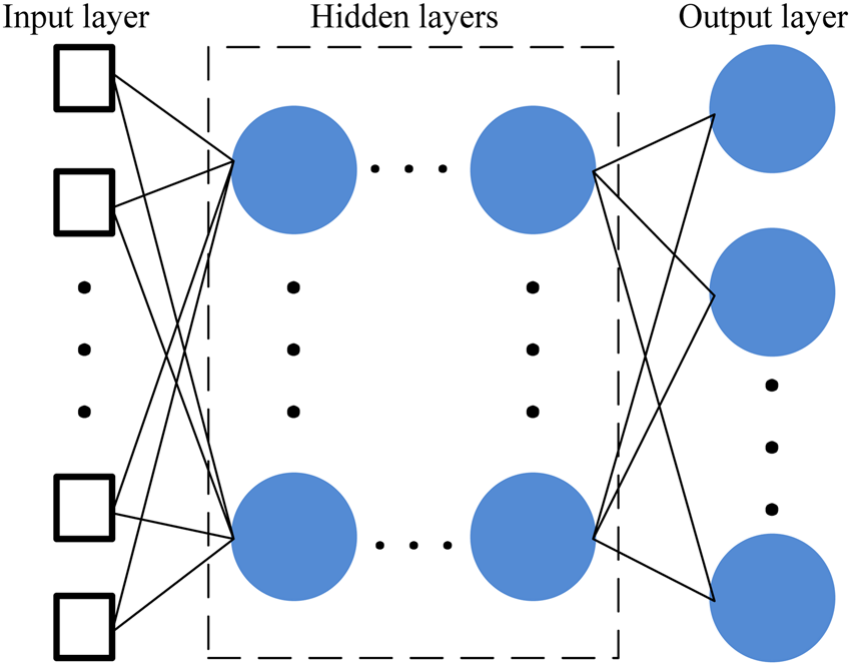
Deep back propagation neural network.

The back propagation (BP) NN generally refers to a feedforward NN. The number of the hidden layers in the BP-NN was usually only three layers at most at the past. However, in our work, the number of the hidden layers is more than three layers, so the BP-NNs we use are deeper in the hidden layers. Hence, we call them DBP-NNs. The deeper the hidden layers, the stronger the ability to learn the features of data. The structure of a DBP-NN is shown in Fig. 3, which consists of an input layer, multiple hidden layers and an output layer. Each layer is composed of many neurons except for input layer. Each neuron consists of inputs, weights, a bias, and an activation function. The output of each neuron is defined as

11 $${y}_{o}={f}_{o}({\boldsymbol{wx}}+b),$$

where ***x*** is the inputs, ***w*** is the weights, *b* is the bias, *f*_*o*_ is the activation function, and *y*_*o*_ is the output of the neuron. A satisfied NN can be obtained by adjusting the weights and biases.

#### Cost function

It has been proved that NN, whose cost function is mean squared error (MSE) function, can estimate posterior probabilities for finite samples with high accuracy^16,17^. For the DBP-NNs, we take the MSE function as the cost function, which is defined as

12 $$E=\frac{1}{n}\,{\sum }^{}\,{({\boldsymbol{y}}-{{\boldsymbol{y}}}_{{\boldsymbol{NN}}})}^{2},$$

where *n* is the number of groups of training set data, ***y*** is the target outputs (DRs in this paper), and ***y***_***NN***_ is the outputs of DBP-NN during training.

### RBF-NN

**Figure 4:**
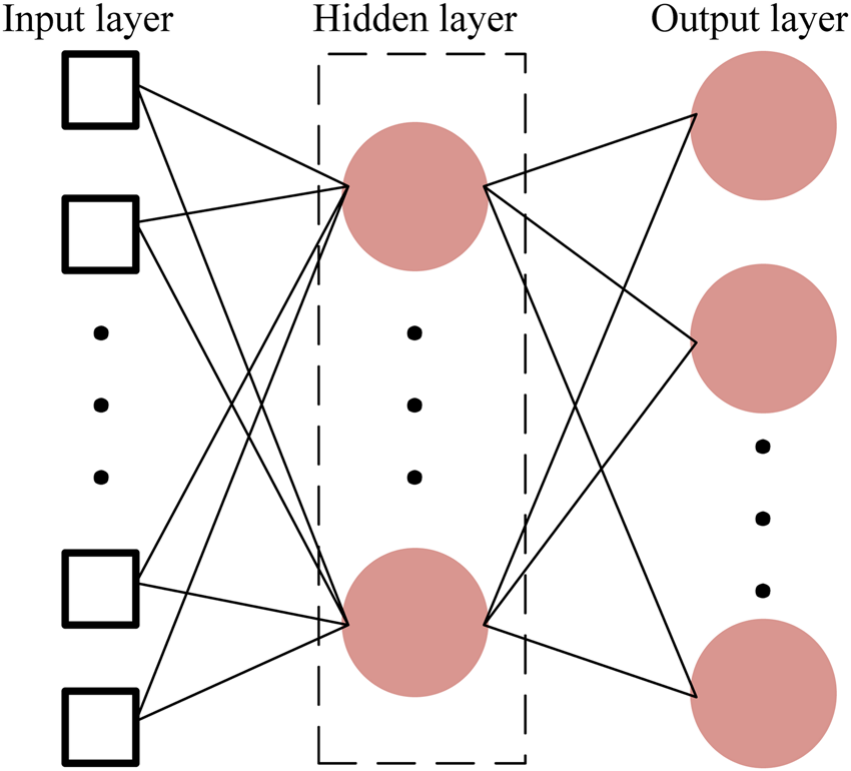
Radial basis function neural network.

Radial basis function neural network (RBF-NN) can approximate arbitrary nonlinear functions. With good generalization ability, it is able to learn complicated laws in a system, and its learning efficiency is remarkable. RBF-NN is composed of an input layer, a hidden layer and an output layer, as is shown in Fig. 4. For the RBF-NN, there is no weight connecting between the input layer and the hidden layer, but the weight connecting between the output layer and the hidden layer. RBFs can calculate the distance or similarity between the inputs and the centers of the hidden layer. The farther the distance is or the lower the similarity is, the smaller the activation of a neuron is, and the less obvious its effect is.

#### Linear regression

For the RBF-NNs in this paper, we use linear regression method to calculate the weight between the hidden layer and the output layer. Compared to the gradient descent method, linear regression method saves the training time and its model is simpler. The weights calculated by linear regression method are as follows

13 $${\boldsymbol{\beta }}={({{\boldsymbol{y}}}_{{\boldsymbol{NN}}}^{{\boldsymbol{T}}}{{\boldsymbol{y}}}_{{\boldsymbol{NN}}})}^{-1}{{\boldsymbol{y}}}_{{\boldsymbol{NN}}}^{{\boldsymbol{T}}}{\bf{y}},$$

where ***β*** is the weights, ***y*** is the target outputs, and ***y***_***NN***_ is the outputs of RBF-NN during training.

### Data set

The data set is composed of the training set, the validation set and the testing set, where the data in the training set, the validation set and the testing set are completely different from each other. The parameters, filling fraction $\bar{a}$, shear modulus ratio $\bar{\mu }$ and mass density ratio $\bar{\rho }$, are taken as the input of the NNs, and the first three eigen modes of the corresponding DRs calculated by TMM as the labels. In our work, three cases are considered. For the first case, the training set A, validation set A and testing set A consist of 10, 2 and 2 sets of data respectively, where $\bar{\mu }$ and $\bar{\rho }$ are unchanged and the range of $\bar{a}$ is from 0.3 to 0.75; for the second case, the training set B, validation set B and testing set B consist of 100, 20 and 20 sets of data respectively, where $\bar{a}$ is unchanged and the ranges of $\bar{\mu }$ and $\bar{\rho }$ are respectively from 0.005 to 0.095 and from 0.1667 to 0.5667; for the third case, the training set C, validation set C and testing set C consist of 1000, 100 and 100 sets of data respectively, where the range of $\bar{a}$, $\bar{\mu }$ and $\bar{\rho }$ are respectively from 0.3 to 0.75, from 0.005 to 0,095 and from 0.1667 to 0.5667.

## Results and Discussions

The performances of the trained DBP-NNs and RBF-NNs are tested for three cases, involving geometric parameter changes, physical parameters changes and simultaneous changes, respectively.

The computing platform used in our work is a laptop whose configuration is shown in Table 1.

**Table 1:** Computer configuration.

Classification	Name
CPU	Inter® Core™ i5-5200U CPU @2.20 GHz
RAM	8 GB (DDR3L 1600 MHz)
OS	Windows 10 professional 64-bit

All the programs are written in “Python 3.5”. The DBP-NNs are developed in “Tensorflow”. The function of “time.clock()” is used to calculate the running time of programs.

Here we measure the prediction accuracy by calculating the Euclidean distance (ED) between the predicted DRs and the target DRs. The smaller the Euclidean distance is, the higher the prediction accuracy is. The ED is defined as

14 $$ED=\sqrt{\mathop{\sum }\limits_{i=1}^{n}\,{({y}_{i}-{\tilde{y}}_{i})}^{2}}$$

where, *y*_*i*_ is the target value, ${\tilde{y}}_{i}$ is the predicted value, and *n* is the dimension of DRs.

### The choice of NNs architectures

In this section, the architectures of the two NNs are discussed for three cases. The optimal architectures of the two NNs are determined by comparing the mean errors of the corresponding validation sets.

#### Choosing the DBP-NNs architectures

For one-parameter prediction, the following four architectures of the DBP-NNs are compared:

DBP-1-1: 1-30-30-30-303

DBP-1-2: 1-30-30-30-30-303

DBP-1-3: 1-30-30-30-30-30-303

DBP-1-4: 1-30-30-30-30-30-30-303

For two-parameter prediction, the following four architectures of the DBP-NNs are compared:

DBP-2-1: 2-300-100-100-100-303

DBP-2-2: 2-300-100-100-100-100-303

DBP-2-3: 2-300-100-100-100-100-100-303

DBP-2-4: 2-300-100-100-100-100-100-100-303

For three-parameter prediction, the following four architectures of the DBP-NNs are compared:

DBP-3-1: 3-600-300-100-100-303

DBP-3-2: 3-600-300-100-100-100-303

DBP-3-3: 3-600-300-100-100-100-100-303

DBP-3-4: 3-600-300-100-100-100-100-100-303

where “1”, “2” or “3” is the dimension of the input layer, “303” is the number of the neurons in the output layer, and others are the number of the neurons in the hidden layers.

**Figure 5:**
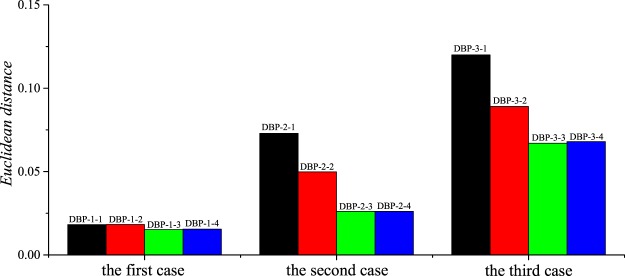
Comparisons of the mean errors of the corresponding validation sets for different DBP-NNs.

Figure 5 gives the mean errors of the validation sets of the DBP-NNs under three cases. It can be seen that for one-parameter prediction, the mean error of “DBP-1-3” is smaller than others, for two-parameter design, “DBP-2-3” and “DBP-2-4” have a similar accuracy, but “DBP-2-3” has less hidden layers than “DBP-2-4”, and for three-parameter design, “DBP-3-3” is the best choice.

#### Choosing the RBF-NNs architectures

Similar to the previous section, for one-, two- and three-parameter prediction, the following twelve architectures of the RBF-NNs are respectively compared:

RBF-1-1: 1-5-303

RBF-1-2: 1-10-303

RBF-1-3: 1-15-303

RBF-1-4: 1-20-303

RBF-2-1: 2-50-303

RBF-2-2: 2-100-303

RBF-2-3: 2-150-303

RBF-2-4: 2-200-303

RBF-3-1: 3-200-303

RBF-3-2: 3-400-303

RBF-3-3: 3-600-303

RBF-3-4: 3-800-303

**Figure 6:**
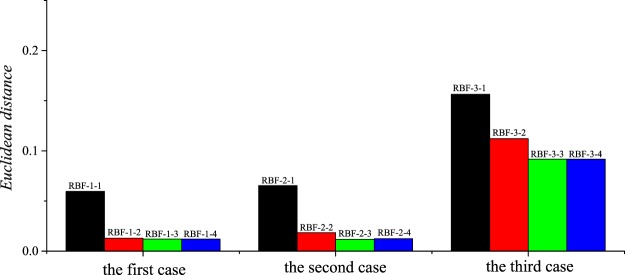
Comparisons of the mean errors of the corresponding validation sets for different RBF-NNs.

Figure 6 gives the mean errors of the validation sets of the RBF-NNs under three cases. It can be seen that “RBF-1-3”, “RBF-2-3” and “RBF-3-3” are the optical architectures for the first, second and third cases, respectively.

### One-parameter prediction

**Figure 7:**
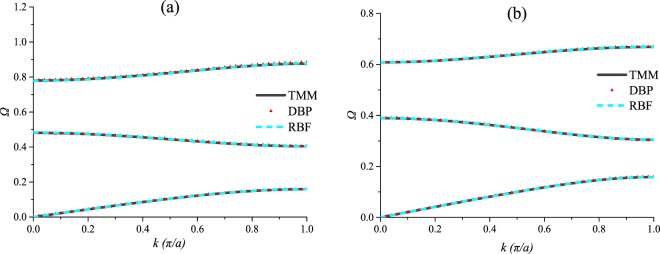
Predictions by the deep back propagation neural network and the radial basis function neural network for the testing set A.

**Figure 8:**
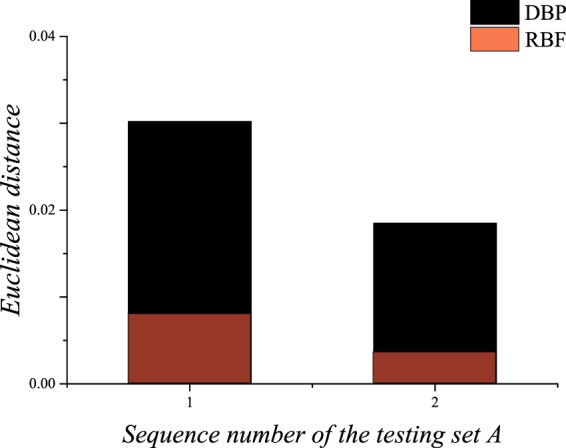
The Euclidean distance between the predicted DRs and the target DRs for the testing set A.

In this section, the DRs of PCs with different filling fractions are predicted, and the data set is composed of the training set A, the validation set A and the testing set A. In this section, the architecture of the DBP-NN is “DBP-1-3”, and the architecture of the RBF-NN is “RBF-1-3”. The predicted DRs of the testing set A are shown in Fig. 7, and the prediction accuracies are shown in Fig. 8. It can be seen that the predicted DRs are in good agreement with the target values. The two NNs exhibit good performances for predicting the DRs of PCs with different filling fractions, but the RBF-NN is better.

### Two-parameter prediction

**Figure 9:**
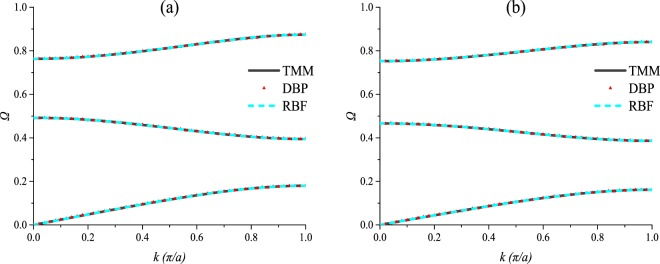
Predictions by the deep back propagation neural network and the radial basis function neural network for the testing set B.

**Figure 10:**
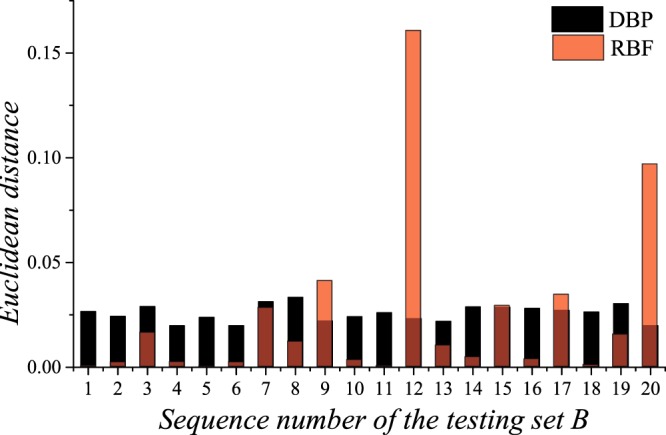
The Euclidean distance between the predicted DRs and the target DRs for the testing set B.

In this section, the DRs of PCs with different shear modulus ratios and mass density ratios are predicted, and the data set is composed of the training set B, the validation B and the testing set B. Due to space limitations, only 2 sets of predictions are shown in Fig. 9 as examples, but the prediction accuracies of the 20 sets are all shown in Fig. 10. Here, the architecture of the DBP-NN is “DBP-2-3”, and the architecture of the RBF-NN is “RBF-2-3”. It can be noticed from Fig. 9 that for two-parameter, both the two NNs present satisfied predictions with high precision. From Fig. 10, it can be seen the DBP-NN has more stable performance, and the RBF-NN has several relatively large errors, although its most errors are very small.

### Three-parameter prediction

**Figure 11:**
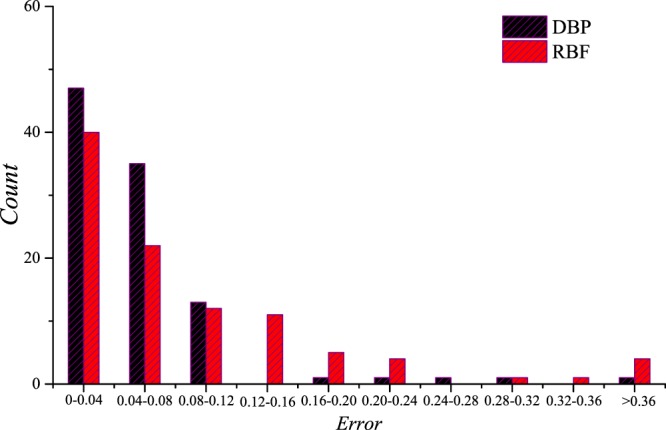
Predictions by the deep back propagation neural network and the radial basis function neural network for the testing set C.

**Figure 12:**
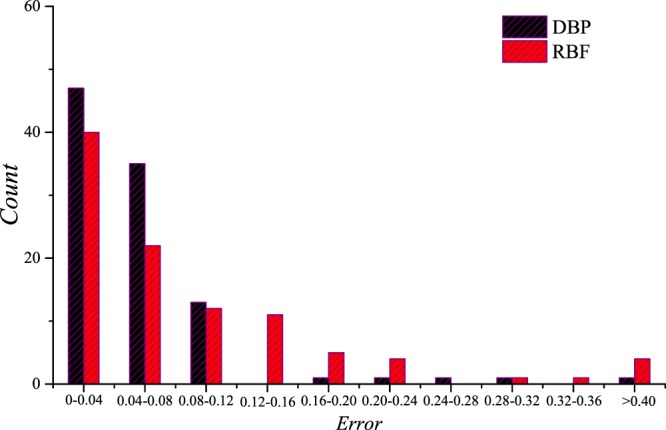
The error statistics of the predicted results for the testing set C.

Predictions of the DRs for different filling fractions, shear modulus ratios and mass density ratios are carried out, and the data set is composed of the training set C, the validation set C and the testing set C. Only 2 sets of predictions are shown in Fig. 11 as examples, while the error statistics of the predicted results of the testing set C are shown in Fig. 12. In this section, the architecture of the DBP-NN is “DBP-3-3”, and the architecture of the RBF-NN is “RBF-3-3”. It can be seen that the performances of the two NNs are still remarkable, but the DBF-NN performs much better.

Comparison among DBP-NNs, RBF-NNs and TMM are given in Table 2. It can be noticed that the time required by NNs is extremely short, and the prediction accuracy is remarkable. For one-parameter prediction, the RBF-NNs are superior to the DBP-NNs on training time, prediction accuracy and simplicity of the model. For two-parameter prediction, the RBF-NN has a smaller mean error, but the DBP-NNs are better than the RBF-NNs in terms of the performance stability from Fig. 10. For three-parameter prediction, the DBP-NN is a better choice because of its high accuracy and stability. In terms of calculation time, TMM is hundreds of thousands of times DBP-NNs and RBF-NNs

**Table 2:** Comparison among the deep back propagation neural networks, radial basis function neural networks and transfer matrix method.

Parameters considered	Method	Model training time(s)	Calculation time(s)	Mean of ED
$\bar{a}$	DBP	19.76	2.27E-02	0.02
	RBF	0.01	4.71E-04	0.01
$\bar{\mu }$, $\bar{\rho }$	DBP	168.34	8.45E-03	0.03
	RBF	1.03	4.45E-03	0.02
$\bar{a}$, $\bar{\mu }$, $\bar{\rho }$	DBP	789.34	2.46E-03	0.06
	RBF	61.92	1.19E-02	0.09
	TMM		511.23	

Model training time refers to the time training a neural network needs; Calculation time refers to the average time taken by the trained neural networks or the TMM to calculate the DRs of a PC; Mean of ED is the mean of the accuracies of the corresponding testing set.

## Conclusions

The deep back propagation neural networks (DBP-NNs) and the radial basic function neural networks (RBF-NNs) are trained to predict the dispersion relations (DRs) of one-dimensional (1D) phononic crystals (PCs) for three different cases in our work. The results show that both the DBP-NNs and the RBF-NNs can predict the DRs of PCs with rather short time and high accuracy. For one-parameter prediction, the RBF-NNs are superior to the DBP-NNs on training time, prediction accuracy and simplicity of the model. For two-parameter prediction, the DBP-NN has more stable performance. For three-parameter prediction, the DBP-NN is a better choice because of its high accuracy and stability.

This paper confirms the feasibility and superiority of using NNs to predict the DRs of PCs. It is the fact that 2D and 3D problems are more complex in design and calculation, consuming much more time and computer memory. Therefore, the application of NNs to the design and analysis of 2D and 3D PCs and metamaterials will be of great significance. How to design a suitable NN to solve the problems of PCs and metamaterials, especially their inverse design problems, will be the difficulty and focus of the future research. The present work provides a useful reference for the related investigations in the future.
