# Author Correction: Predicting the Dispersion Relations of One-Dimensional Phononic Crystals by Neural Networks

**DOI:** 10.1038/s41598-020-57445-5

**Published:** 2020-01-15

**Authors:** Chen-Xu Liu, Gui-Lan Yu

**Affiliations:** School of Civil Engineering, Beijing Jiaotong University, Beijing, 100044 China

Correction to: *Scientific Reports* 10.1038/s41598-019-51662-3, published online 25 October 2019

In this Article, Figure 11 is a duplication of Figure 12. The correct Figure 11 appears below as Figure 1.

**Figure 1:**
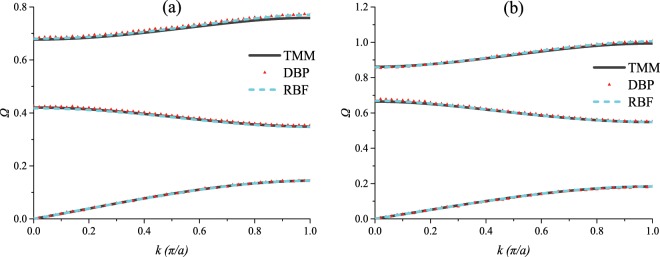
.

